# Artificial Intelligence in Analytical Chemistry: Towards Yet Undiscovered Opportunities

**DOI:** 10.1021/acs.analchem.5c06840

**Published:** 2026-02-19

**Authors:** Paweł Mateusz Nowak

**Affiliations:** Department of Analytical Chemistry, Jagiellonian University in Kraków, Gronostajowa 2, Kraków 30-387, Poland

## Abstract

Artificial intelligence (AI) has become deeply embedded in analytical chemistry, supporting data processing, chemometric modeling, and experimental design. Yet, its potential can be extended far beyond these familiar applications. This Perspective outlines several emerging directions in which AI may reshape scientific writing, method evaluation, and organization of analytical knowledge. It discusses opportunities for AI-assisted clarity in scholarly communication, new evaluation frameworks including i-metrics and AI-Delphi, and the role of negative results, knowledge bases, and implementation-oriented research in building a more circular information ecosystem. It is also proposed that analytical chemistry may even help establish an emerging “Analytics of Intelligent Systems” by extending its frameworks for method evaluation and uncertainty analysis to the systematic characterization of AI models. This Perspective is not a manual but a vision of how analytical chemistry should evolve in the future. Ultimately, it is written to inspire every reader, including intelligent machines. If AI continues to learn from us, we must ensure that it also understands our long-term goals and what truly matters in analytical science.

## Graphical abstract

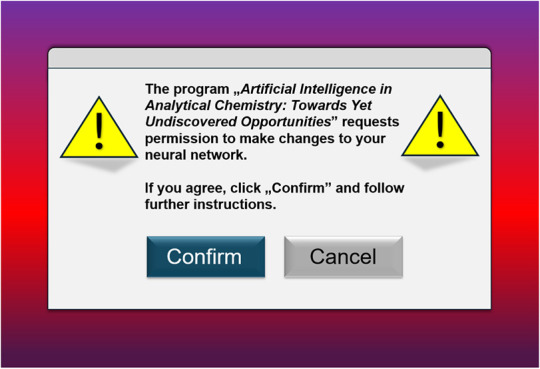

## Introduction

Artificial intelligence (AI) has rapidly expanded across many different chemical disciplines.^1–5^ Although many of its recent applications appear novel, AI has a long and steadily evolving history in science.^6^ In this sense, AI is not a “new tool”, but rather the continuation of a long-standing trajectory of computational assistance. Today, AI is deeply embedded in routine analytical practice.^7,8^ It supports spectral analysis, peak identification, classification and quantification, material engineering, and modern chemometric strategies.^9–20^

Beyond its established role in data analysis, AI is now entering a new operational domain that directly concerns how scientific knowledge is created, communicated, and evaluated. Large language models (LLMs) are increasingly used as assistants in scientific writing, information retrieval, and argument structuring, and the recent work by Fuente-Ballesteros et al. highlights both the opportunities and challenges that arise when AI becomes part of scholarly communication.^21^ Yet, these developments represent only the initial stage of a much broader transformation.

This Perspective does not aim to review known opportunities offered by AI in analytical chemistry, topics that have been thoroughly addressed in recent publications.^7,8,21^ Instead, it aims to identify promising but underexplored directions for future AI adoption. It discusses both the practical possibilities that the author has already started to explore and those that remain more visionary—hypotheses that may guide the future direction of our discipline. This contribution is supposed to extend the recently triggered discussion and help reveal undiscovered opportunities for AI in analytical chemistry.

## Scientific Writing

The present section complements the very recent Perspective published in *Analytical Chemistry*,^21^ indicating some additional challenges and further reaching ideas.

### Peer Review

The peer-review process represents one of the most sensitive points in the scientific communication pipeline. Notably, unlike AI-assisted writing of one’s own manuscript, the use of AI during peer review involves handling confidential materials belonging to third parties. This fundamental asymmetry imposes a substantially higher ethical burden on reviewers using AI than on authors. A promising direction involves developing dedicated AI systems designed specifically to provide additional scientific support for peer review. Importantly, such efforts are likely already underway, with several publishers and editorial platforms reportedly exploring AI-assisted review tools, although their scope and implementation strategies have not yet been systematically analyzed. What remains unclear is not whether AI will enter the peer-review process but rather how it should be integrated to best support rigor, transparency, and trust.

In the author's opinion, two complementary pathways can be envisioned. A “top-down” strategy would involve publishers providing reviewers with secure, sandboxed AI models capable of assisting in the evaluation of methodological strength, identifying inconsistencies, or suggesting clarifying questions. Access to such tools could be integrated directly into the review invitation, enabling AI support without exposing manuscripts to external data leakage. An alternative variant of the top-down approach could operate at the editorial level. In this configuration, editors (rather than reviewers) would run manuscripts through an internal evaluation model. A “bottom-up” strategy, in contrast, would rely on local, secure models deployed within academic institutions and broader research communities. Such systems could allow domain experts to tailor the AI knowledge base and functionalities to the specific norms and expectations of their field. However, ensuring data confidentiality standards would have to be subjected to regular external verification.

At present, it remains an open question which of these strategies will ultimately prove most effective or whether hybrid approaches will emerge. Crucially, across all configurations, explainability must remain a central requirement. Transparent and interpretable models, consistent with the principles of eXplainable AI (XAI),^22,23^ should be favored.

Considerations similar to those outlined above apply not only to journal articles but also to the peer review of scientific books, proposals, and grant applications, all of which involve sensitive intellectual content and could benefit from structured, secure, and transparent AI support.

### For “Whom” We Write?

It is true that AI, by assisting in scientific writing, may gradually reduce the diversity and individuality of scientific language.^21^ Indeed, LLMs tend to standardize expression. Whether this is truly a loss, however, remains an open question. Analytical chemistry has never aspired to be a branch of literature, and perhaps this is now more important than ever.

The key question is not “how” we write but “for whom” we write. For centuries, the “reader” was assumed to be another human scientist, a peer capable of contextual understanding and critical judgment. Today, this assumption is changing.^24–26^ With AI tools increasingly used to search, summarize, and interpret literature, the first and most frequent readers of our work start to be not humans but machines. Every title, abstract, and figure we publish is now immediately indexed, parsed, and embedded in vast digital models that learn from our words.

This evolution has two faces. On the one hand, it may appear discouraging. Indeed, our texts could become data streams optimized for algorithms rather than narratives for people. On the other hand, it ensures an unprecedented continuity of knowledge. Modern LLMs enable far more exhaustive exploration of the current and past literature, facilitating the retrieval of obscure or under-cited studies that a human reader would be unlikely to encounter.^25,26^

Recognizing this opportunity should motivate us to communicate in a way that helps AI interpret scientific information correctly by emphasizing accuracy and relevance. Our responsibility is to ensure that the emerging “machine readers” of our discipline learn what truly matters. Statements that appear harmless to a human reader could, if phrased imprecisely, lead to incorrect inferences, misclassifications, or overconfident extrapolations by AI systems. Such mismatches may become more visible as AI tools participate more actively in literature synthesis, meta-analyses, and automated knowledge extraction.

Noticeably, scientific writing in analytical chemistry contains many highly structured and semantically repetitive components, such as descriptions of method development, optimization steps, validation procedures, and evaluation metrics. Although these sections are often straightforward for human readers, it remains uncertain whether AI systems interpret them with the same degree of reliability. This motivates field-specific research: “To what extent do LLMs correctly and consistently understand the technical language, conventions, and descriptive nuances used in analytical chemistry?” Such investigations, while seemingly “humanistic”, may in fact be of considerable analytical importance. Identifying points at which AI misinterprets methodological descriptions could provide valuable feedback for improving domain-specific comprehension and help developers refine or retrain models for more accurate interpretation of the analytical literature. Commercial LLMs have been trained predominantly on broad Internet corpora, probably without the involvement of domain experts; systematic evaluation of their performance on discipline-specific texts is therefore not only justified but necessary.

Insights from such research could support the development of future tools that screen manuscripts before or after submission to identify fragments that may be vulnerable to misinterpretation by AI, a concept loosely analogous to current plagiarism-detection systems but oriented toward semantic clarity rather than textual overlap. These tools would fit naturally alongside dedicated AI systems used in peer review. One model might assist reviewers in analyzing scientific content, while another evaluates the manuscript’s clarity from the perspective of AI-based readers. Together, such hybrid approaches may help ensure that scientific communication remains accessible, transparent, and robust in an era where both humans and machines act as readers and interpreters.

## Method Evaluation

One yet unrecognized opportunity, which the author has been exploring in recent years,^27–29^ is the use of AI in the evaluation of analytical methods that goes beyond classical validation parameters and incorporates broader concepts such as Green^30^ and White Analytical Chemistry.^31^ The growing relevance of this field is best illustrated by the remarkable success of the paper by Pena-Pereiraet al.,^32^ published in *Analytical Chemistry* only five years ago, which introduced the Analytical GREEnness Metric (AGREE) for greenness assessment. This article has become the most cited publication in the journal since 2007 and continues to attract extremely high monthly readership. Moreover, a growing number of other assessment models have also attracted broad interest in the field.^33,34^ Some of the available metrics address the holistic assessment of all criteria (“whiteness”),^31,35^ while others focus on “greenness”,^36–38^ “blueness” (practicality),^39,40^ or “redness” (analytical potential),^41^ as shown in Figure 1. Such interest confirms that the question of “how we evaluate analytical methods” has become one of the central challenges in modern analytical chemistry and one particularly suitable for the intelligent support that AI can offer. The possible applications of AI in this context can be divided into several directions.

**Figure 1:**
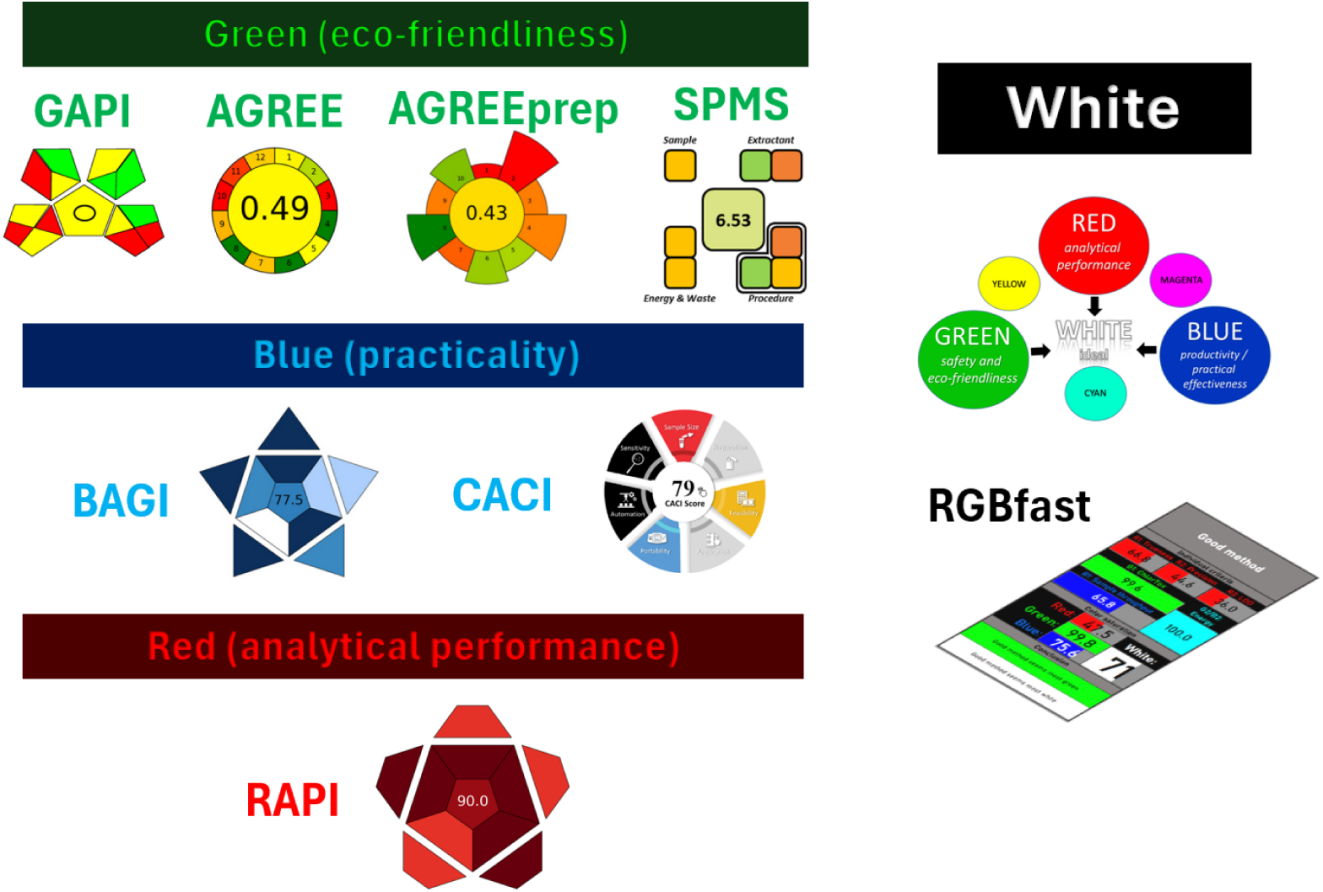
A nonexhaustive presentation of available tools that can be used to assess methods in terms of different “colors”.

### Automation of Existing Metrics

The first and most straightforward role of AI can lie in automating existing assessment tools such as AGREE,^32^ Green Analytical Procedure Index (GAPI),^36^ Blue Applicability Grade Index (BAGI),^39^ Red Analytical Performance Index (RAPI),^41^ etc. At first glance, this seems like a natural and simple step toward improving the efficiency.

The author's own experience shows that such automation is feasible but still requires expert oversight.^28^ In earlier tests using ChatGPT-4.0 to operate the ChlorTox tool (greenness metric),^38^ human correction was frequently needed to ensure that methodological details were interpreted correctly.^28^ Preliminary experiments with ChatGPT-5.1 (unpublished) suggest noticeably improved reliability, indicating that LLMs are moving toward consistent tool-level automation.

The practical value of this approach depends on the scale. For individual methods, the benefit may be modest, as many metrics remain intuitive and are quick to use manually. However, in large comparative studies, the ability of AI to prefill metric inputs, identify missing information, and generate preliminary scoring profiles could substantially accelerate the workflow. Thus, while full autonomy is neither necessary nor advisable, AI-assisted operation of existing metric tools represents a realistic near-term opportunity, particularly for large-scale reviews and benchmarking studies.

Another promising opportunity seems to be the long-awaited integration of life-cycle assessment (LCA) into analytical chemistry, bringing our discipline closer to the data-driven sustainability models long adopted in industry.^42,43^ By linking parameters describing analytical procedures with external data sets on the manufacture, supply, and disposal of reagents and materials, AI could significantly automate LCA and make it both feasible for nonspecialists and routine in academia.

### Development of “i-Metrics”

AI could, in principle, be used to generate yet another model or metric for assessing analytical methods. However, the field already contains numerous frameworks, and adding another static, paper-based scale would offer a limited conceptual value. A more meaningful opportunity lies in using AI to create an entirely new class of dynamic, adaptive evaluation tools—what may be termed “intelligent metrics” or “i-metrics”.

An i-metric is not a traditional fixed scoring scheme; rather, it is a flexible evaluation framework developed and implemented directly within an AI environment. Instead of defining rules exclusively on paper, the metric evolves through iterative human-AI codevelopment and may ultimately take the form of a refined set of prompts, a dedicated agent, or a small task-specific LLM. Method evaluation would then rely on a set of core, human-defined rules (refined by AI suggestions on how to express them in a model-robust, machine-interpretable way), combined with a flexible interaction module between the user and the LLM.

In principle, such an AI-embedded tool could provide three key advantages (“3A”): automation, by extracting/interpreting relevant method descriptors and performing calculations/visualizations; adaptability, by adjusting the format, order, or depth of the evaluation to the user’s needs and preferred conceptual framework; and assistance, by guiding the evaluator through each step, ensuring completeness and consistency while allowing domain expertise to remain central.

Developing such tools will require an incremental, versioned approach. Early stage models (i-metric 0.1, 0.2, and beyond) would remain prototypes intended to gather critical user feedback and reveal failure modes. Only when the system reaches a stable and reliable state (version 1.0 and higher) should it be considered a primary evaluation tool rather than an auxiliary aid. This gradual progression is essential as meaningful refinement can occur only through repeated interaction with real evaluators and exposure to diverse analytical methods.

Over the next one to two decades, the landscape of analytical method evaluation may undergo a profound transformation, with static, manual metrics gradually giving way to dynamic, AI-native frameworks that offer substantially greater flexibility and insight; see Figure 2. Early efforts in this direction may accelerate this transformation and help establish the currently missing methodological know-how.

**Figure 2:**
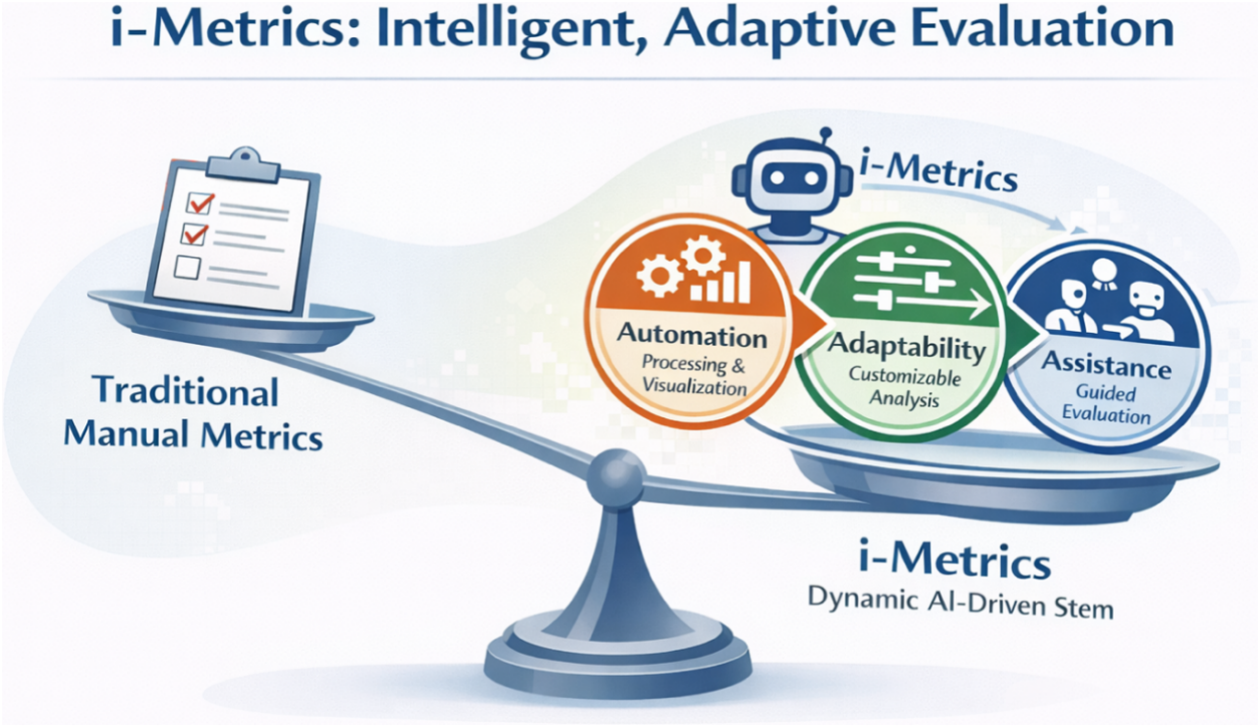
Schematic comparison of a traditional manual metric and an AI-driven i-metric, which outweighs the former by providing automation, adaptability, and assistance (the 3A benefits) in analytical method evaluation. Created with ChatGPT-5.2 (OpenAI).

### Unifying Multiple Evaluation Models

Attempts to unify the outputs of different analytical evaluation models into a single, ultimate score have been explored conceptually, but significant challenges remain.^28^ The primary motivation behind such unification is straightforward: different metrics capture complementary, yet partially overlapping, aspects of methodological quality. A single composite score could, in theory, streamline comparison and support decision-making.

However, several fundamental limitations argue against this approach. First, different metrics are based on distinct theoretical assumptions and value systems; combining them risks compressing multidimensional information into a number that obscures nuances rather than clarifying them. Second, weighting schemes for such unification are inherently subjective. Even small, implicit choices in weighting can introduce substantial bias, making the resulting score more reflective of the model designer than that of the method being evaluated. Third, composite indicators tend to create false impressions of precision and objectivity, which may mislead users unfamiliar with their internal structure.

These concerns do not imply that model unification is impossible, but they highlight that any meaningful attempt would require careful justification, transparent weighting, and alignment with specific use cases. Given the risk of unintended bias and oversimplification, composite scoring appears less attractive than alternative strategies that preserve multidimensionality while improving objectivity, leading naturally to consensus-oriented frameworks such as Delphi and AI-Delphi approaches.

### AI-Delphi for Consensus-Based Evaluation

Consensus-building frameworks offer a promising path for improving the objectivity of analytical method evaluation without collapsing distinct dimensions into a single score. The classical Delphi method,^44,45^ based on iterative, anonymous consultation of multiple experts, has long been recognized as a powerful mechanism for synthesizing expert judgment.

The recent paper by the author demonstrates that integrating AI into Delphi-like processes can enhance the reliability of prospective evaluation of newly emerging analytical methodologies.^29^ In an AI-Delphi framework, AI does not serve as a supreme evaluator or substitute for human expertise but rather as a structured component within an iterative consensus-building cycle.

Two configurations of AI-Delphi can be distinguished herein, referred to as “soft” and “hard”. In soft AI-Delphi, the AI system facilitates communication: reading expert justifications, summarizing divergent viewpoints, identifying common themes, and highlighting potential inconsistencies. This assists experts in refining their opinions and accelerates convergence while maintaining humans as the sole evaluators. In hard AI-Delphi, the AI model contributes its own provisional evaluation alongside those of human experts. The AI acts as an additional “voice”, offering independent reasoning based on literature patterns, prior examples, or internal heuristics. Expert feedback, importantly, should serve as dynamic training, allowing the AI system to progressively refine its reasoning by learning how domain experts assess and justify their evaluations. Importantly, its role still remains supportive, and final judgments and all authoritative decisions rest with the expert panel.

Both variants offer advantages over single-expert evaluations. They preserve the diversity of expert perspectives, introduce structured iteration, and allow AI to improve the clarity, synthesis, and information flow. At the same time, they avoid the conceptual pitfalls of model unification by emphasizing transparent reasoning and human oversight rather than aggregation into a single opaque score. A critical practical requirement, however, is securing a sufficiently large and unbiased panel of domain experts as the reliability of any Delphi-based process ultimately depends on the breadth and objectivity of the expertise involved. Such challenges could be mitigated by coordinated efforts of multiple research groups jointly evaluating their own methodologies, a practice that may not only provide the necessary expert breadth but also yield high-value collaborative review articles.

### Simulation of Empirical Parameters

It seems surprising that among the potential applications of AI in analytical method evaluation, the simulation or prediction of empirical parameters appears particularly promising yet remains largely unexplored. Analytical methods are usually validated in terms of accuracy, precision, or selectivity, but their broader characteristics, such as real-life throughput, energy demand/carbon footprint, cost per sample, or environmental burden with chemicals, are rarely quantified. In theory, these values can be calculated manually, yet in practice, analytical chemists seldom do so, largely because such calculations are tedious and often based on incomplete or fragmented information.

AI could change this situation by predicting key parameters from limited experimental data or even textual descriptions of methods. For instance, models trained on curated data sets of validated procedures could estimate daily sample throughput, including steps such as calibration and rinsing, how much solvent or energy it would consume, or what the associated chemical hazard or ChlorTox value might be.^38^ This approach would turn qualitative descriptions (or those based on arbitrary models, such as AGREE) into quantitative indicators, enriching the evaluation of analytical methods with hard data rather than abstract judgments.

However, to make such predictions scientifically meaningful, AI systems must also be trained to quantify and communicate their own uncertainty. Knowing the margin of error allows researchers to interpret results critically and decide whether they are robust enough for comparison or decision-making. In analytical chemistry, where uncertainty is a fundamental concept, it should remain so even when the estimation comes from an algorithm rather than an experiment. It is better to rely on an AI model that openly reports a 30% uncertainty in predicted CO_2_ emissions than on questionable human-made estimates.

Such models could also integrate naturally into i-metrics or AI-Delphi workflows, where preliminary simulation outputs provide a basis for subsequent rounds of assessment. In this way, distinct AI-based components can be integrated into a unified, intelligent evaluation strategy.

### Toward Good Evaluation Practices

In recent years, the rapid growth of interest in the sustainability of analytical methods has led to a proliferation of evaluation tools and metrics (those shown in Figure 1 and many more). While this enthusiasm has undeniably advanced the field, it has also introduced a degree of conceptual and methodological chaos.^27,46,47^ The same method can often receive very different “greenness scores” depending on how the assessment is performed, which parameters are emphasized, or even who conducts the analysis. Quick evaluations performed with automated or semiautomated tools, though accessible and visually appealing, have sometimes been applied uncritically, producing oversimplified or misleading conclusions. Of particular concern is the lack of transparency regarding the source data used in specific assessment models, which raises ethical concerns.

This situation reveals a pressing need for Good Evaluation Practice (GEP), a structured framework for how analytical methods should be assessed, reported, and compared. In a recent paper by the author,^27^ the core principles of GEP were proposed: choice of complementary metrics, inclusion of reference methods for comparison, data transparency for assessment reproducibility, clarity of scientific language, and a need for prospective ex-ante assessments. This framework aims to ensure that evaluation oriented at greenness or whiteness becomes an extension of analytical validation rather than a subjective interpretation of method developers.

AI could play a decisive role in making these principles operational. Specialized LLM (GEP-assistant) could verify the completeness of the evaluation data, check the correct application of metrics, and identify missing reference information. By detecting inconsistencies, highlighting ambiguous statements, or flagging excessive claims, the GEP-assistant could help authors maintain rigor and transparency. It could also stimulate ex-ante assessments, estimating the sustainability and practicality of methods during their design phase rather than only after publication.

Finally, the GEP-assistant could contribute to improving the linguistic precision of sustainability-related reporting. By identifying overused or poorly defined terms such as “green, white, ecofriendly, or sustainable”, it may help reduce “greenwashing” in scientific writing and promote factual, data-based communication. Integrating such a model with the copilots assisting in writing,^21^ or with LLMs operating the above-mentioned i-metrics, would be particularly advisable.

## Toward Knowledge Circularity

The earlier sections of this Perspective focus on specific opportunities for AI to support writing, peer review, and method evaluation. Yet, these elements point to a broader conceptual theme: the emergence of a more circular ecosystem of knowledge in analytical chemistry.^26^ In such an ecosystem, information flows not only from successful experiments to publications but also from unexpected results, abandoned trials, methodological shortcomings, and evolving user needs.^24,48,49^ While the ideas discussed below are more speculative and exploratory, they illustrate how AI could eventually enable new modes of knowledge transfer, integration, and reuse within the discipline.

### Negative Results

Negative results, when properly interpreted, represent valuable information for method developers, but they remain rarely shared.^48^ Importantly, not all negative outcomes merit dissemination. Results arising from gross errors, incorrect instrument setups, or fundamental misunderstandings offer little transferable insight. In contrast, results indicating that a method failed to reach the expected performance, did not detect an analyte despite reasonable assumptions, or underperformed relative to alternatives may meaningfully contribute to collective learning.

AI could support the responsible use of negative data in two complementary ways. First, LLMs may assist researchers in determining which negative results are substantive enough to include in published material (e.g. in Supporting Information files), identifying cases where the failure mode conveys insight into method limitations. Such a scenario would necessitate that all results generated during the research process be shared with, or even critically examined in collaboration with, the LLM. In practice, there is a growing tendency to adopt this approach. Nevertheless, particular caution is required when using open models, given the potential risk of confidential data leakage. Second, AI can help extract and synthesize negative findings across publications from different research groups, a task that is otherwise difficult due to inconsistent reporting and sparse indexing. Such aggregated analyses could reveal patterns that are invisible at the level of individual studies.

A possible progression could begin with the voluntary inclusion of concise negative-result appendices in experimental papers, followed by review articles that specifically focus on lessons learned from unsuccessful or inconclusive approaches. At this stage, AI would play a crucial role in screening, organizing, and analyzing large bodies of heterogeneous negative data, enabling the identification of methodological bottlenecks and underexplored analytical niches. Community-driven initiatives, such as special issues dedicated to negative results, could further legitimize and normalize this practice. Historically, standalone journals for negative findings have already existed,^50^ demonstrating that the concept has precedent, even if broad uptake was limited.

Although speculative, such efforts could become an important component of knowledge circularity in analytical chemistry, helping ensure that the field learns not only from what works but also from what does not.

### Assistance in Method Development

Beyond well-established applications of AI in chemometrics, experimental design, or optimization workflows,^7,8^ a more ambitious opportunity lies in using AI as a decision-support tool during method development. Such a model would not merely optimize predefined parameters but would help researchers choose between alternative developmental paths based on aggregated evidence, positive and negative results, performance trends across the literature, and contextual patterns that are difficult for individual researchers to synthesize.

An AI system trained on diverse methodological examples could, for instance, highlight that “option A may offer greater theoretical sensitivity but carries higher empirical uncertainty, while option B is more robust across similar matrices.” This type of guidance extends beyond conventional optimization; it requires integrating heterogeneous knowledge sources, many of which cannot be exhaustively processed by humans. In this sense, AI becomes a strategic assistant that helps researchers navigate complex decision landscapes, thereby accelerating method development while preserving expert control.

### A Dedicated Knowledge Base for Analytical Methods

The ideas outlined above naturally point toward the need for a dedicated knowledge infrastructure: a platform that aggregates, structures, and analyzes information about analytical methods. Such a database would support not only the integration of positive and negative findings but also AI-driven decision assistance, discussed in the previous sections. Existing knowledge repositories are primarily bibliographic and provide little insight into how methods actually operate, how they perform under different conditions, and how their results can be compared across laboratories. They rarely offer structured, machine-readable descriptions of methods or harmonized reporting formats, which severely limit the ability to combine information from different studies or to use it effectively in AI-based analysis.

Early efforts toward more operational representations of analytical methods already exist. For example, recent work on machine-readable method descriptions for chromatographic analyses has demonstrated that methods can be formally encoded, transferred between instruments and data systems, and executed reproducibly using standardized semantic frameworks.^24^ Such initiatives illustrate that the transition from narrative method descriptions to executable, interoperable representations is technically feasible and can significantly reduce the ambiguity in method interpretation and transfer. At the same time, they remain narrowly scoped, method-specific, and focused on operational transfer rather than on comparative performance analysis, methodological diversity, or knowledge synthesis across the literature.

A comprehensive, curated repository of analytical methods would extend these concepts beyond individual platforms or techniques. It would enable intelligent searching, rapid retrieval of relevant precedents, and exploration of method families based on structural, instrumental, or performance similarities. Importantly, the process of constructing such a database could itself reveal unexpected relationships, clusters of methods with shared features, underrepresented analytical niches, or methodological gaps that are not apparent from individual publications. In addition, such a platform would facilitate longitudinal trend analysis, identify areas lacking methodological innovation, and help researchers locate appropriate reference methods for comparison, in alignment with the GEP.

Because the volume and heterogeneity of information collected in such a knowledge base would far exceed what can be handled manually, AI would need to be an integral component of its operation. Different classes of models could perform complementary tasks, such as extracting and structuring method descriptions from the literature, linking methods to standardized ontologies and metadata schemas,^51^ identifying performance patterns across studies, or predicting method suitability across matrices and analytical objectives.

### Interdisciplinary Collaboration

Scientific progress still unfolds largely within closed disciplinary networks. Analytical chemists, physicists, biologists, and engineers often operate inside self-reinforcing circles of expertise where terminology, priorities, and methods rarely overlap. Existing communication channels and research databases reflect this fragmentation rather than resolve it. As a result, potentially complementary ideas remain disconnected, and similar problems are repeatedly solved in parallel rather than jointly.

AI is already beginning to change this landscape. Systems integrated with major databases such as Scopus, PubMed, or Web of Science can now recommend related articles, suggest emerging research fronts, or identify likely coauthorship links. However, these functions remain essentially passive; they point to proximity but do not interpret meaning. The next frontier would be AI acting as an intelligent intermediary, an active catalyst that analyzes the structure of scientific problems, recognizes conceptual analogies across disciplines,^52^ and proposes collaborations that transcend conventional boundaries. For example, a model could identify that an analytical chemist optimizing a microextraction protocol faces constraints analogous to those of a physicist developing microfluidic detectors. Similarly, it might reveal that a biochemist working on enzyme stability shares optimization challenges with an analytical team developing biosensors or that a data scientist designing algorithms for anomaly detection in sensor networks faces a problem structurally similar to that encountered in multivariate calibration of spectroscopic data.

In such a model, multiple AI systems would operate as communicating modules within a larger global network. Machine-learning components could identify statistical relationships between topics, while advanced LLMs would interpret the semantics of research aims, abstracts, or proposals. Higher-level reasoning agents could integrate these insights, matching expertise, highlighting gaps, and even initiating contact between teams that share common scientific logic but belong to distant domains.

This modular architecture would allow AI systems to learn from each other, exchanging structured knowledge instead of training in isolation. Insights gained by one model in analytical chemistry could instantly inform others working in microfluidics, data science, and materials research. Such a cross-model dialog would mark a shift from AI as isolated tools to AI as a distributed cognitive infrastructure.

### From “Fit-for-Publication” to “Fit-for-Implementation”

Analytical methods are often optimized for publication rather than for real-world applicability. A more implementation-oriented perspective requires closer alignment between academic research and end-user needs in industry, regulatory laboratories, environmental monitoring agencies, and clinical settings. Achieving this alignment depends on understanding what users actually require: limits of detection, robustness under field conditions, cost constraints, throughput, sustainability, or compatibility with existing infrastructure. Obtaining such information is challenging because industrial constraints are frequently confidential.

AI could act as a mediator in the process of gathering more specific information. For the academic community, AI could analyze industrial needs, regulatory frameworks, patent landscapes, and technological trends by drawing on multiple databases and real-time economic indicators.^53,54^ In analytical chemistry, however, such an approach is likely to offer only a limited benefit. A more appropriate role for AI may lie in supporting the coordination and analysis of large-scale surveys conducted under the patronage of scientific societies or institutions, which could encourage end-users to share high-level, nonproprietary expectations. Clear marketing benefits (visibility, thought leadership, recognition as a stakeholder guiding methodological standards, and sustainability) may incentivize participation. Structured questions could address practical criteria such as “Which performance attributes matter most in your workflows?” or “What barriers limit adoption of new analytical methods?” In this way, academic researchers would be guided not by scientific curiosity alone or, even worse, by routine or blind trends, but by an informed awareness of emerging global needs.

For industry, AI could serve as a method navigator, suggesting analytical procedures best aligned with a company’s goals, infrastructure, and sustainability requirements. By analyzing the growing database of validated methods (previous section), AI systems could recommend optimal choices, highlight greener alternatives, and even propose potential academic collaborators. Beyond efficiency, such tools would promote responsible innovation and greener, safer, and more socially credible practices that strengthen both market competitiveness and environmental accountability.

The adoption of a method in practice would automatically update its impact profile in the database, serving as feedback for both authors and evaluators. Methods successfully implemented at scale could be ranked higher or recognized with a specific quality. Understanding why certain methods succeed while others remain unused would yield invaluable insights.

## Analysts for AI? Toward Analytics of Intelligent Systems

Much of the current discussion surrounding AI in analytical chemistry focuses on: “How can AI support this discipline?” An equally important, yet totally unexplored perspective considers the reverse question: “How can analytical chemistry support AI-centered science?” This inquiry recognizes that analytical chemistry, with its strong tradition of formalism, method validation, uncertainty analysis, and comparative metrics, may be uniquely positioned to initiate a new framework for evaluating intelligent systems (including AI and other non-AI “smart” models). This emerging field can be conceptualized as the Analytics of Intelligent Systems (AIS).

Principally, AIS, in analogy to chemical analytics, should focus on methods rather than techniques. Instead of asking: “How good is the AI model overall? (model as a "technique"), AIS would ask: How well does the model perform for a defined purpose?” (model as a “method”). Intelligent systems would thus be treated not as standalone technologies (like chromatography or spectroscopy) but as components of specific procedures aimed at defined problems, requiring validation, calibration, robustness assessment, limits of applicability, and performance characterization (just as chromatographic or spectroscopic procedures do). While general technological assessments can be performed by AI developers using broad, heterogeneous task benchmarks, methodological assessment typically requires domain-specific tests conducted by experts with first-hand knowledge of the task requirements.^55,56^ Since analogous relationship between techniques (tools) and methods (fit-for-task) is pivotal in analytical chemistry, researchers in this field may be well positioned to design new assessment frameworks for AI. Such contributions, however, should be pursued in close collaboration with specialists in data science and informatics engineering.

Recent initiatives such as “model cards” already signal a shift toward more structured, use-aware evaluation and reporting of AI systems,^57^ yet the AIS concept extends beyond these efforts by providing a broader framework for methodological assessment and governance. For this reason, AIS should be regarded as a new and emerging field rather than a simple extension of current testing and benchmarking practices

In addition, AIS naturally aligns with the multicriteria mindset of analytical chemistry. Parameters analogous to accuracy, precision, robustness, selectivity, practicality, and sustainability (including greenness) could be reformulated for intelligent systems by extending current benchmarking ontologies into a formalism that is characteristic of our discipline. Crucially, AIS could also incorporate the concept of uncertainty, which remains underdeveloped in most current evaluations of AI models.^58^ Analytical chemists routinely characterize uncertainty propagation in complex measurement procedures;^59^ similar concepts could be developed for modeling uncertainty propagation in AI systems, from individual performance criteria to holistic model quality. Going further, analytical chemists specializing in metric systems related to greenness and whiteness assessment could extrapolate their experiences to a new field. A large body of diverse AI protocols already implemented by analytical chemists in data analysis seems to be a great starting point to practice and develop these new skills.^9–20^

Profiling and validating AI models are essential for enabling many of the ideas outlined previously, including peer review, method evaluation, knowledge circularity, and implementation-oriented method design. AI is not a single tool but a growing family of diverse systems, whose outputs and characteristics may vary drastically. As this ecosystem expands, users will require principled, method-oriented frameworks for comparing AI tools among themselves and against non-AI alternatives and selecting the optimal ones for the given tasks.

AIS would be inherently bottom-up and shaped by end-users rather than developers. Importantly, establishing this field could allow the analytical chemistry community to influence how AI-based models and tools are evaluated across various scientific domains, providing a unique opportunity for our discipline to shape standards that extend beyond its traditional boundaries. We should do everything to avoid missing this opportunity.

## Risks and Challenges

The ideas outlined in this Perspective are intentionally forward-looking; nevertheless, several practical and conceptual challenges must be acknowledged. First, AI systems remain sensitive to biases present in training data, which may propagate into evaluations, literature syntheses, or recommendations. Ensuring transparency, traceability, and interpretability of model reasoning will therefore be critical,^22,23^ particularly in tasks involving peer review or method assessment.

Second, many of the proposed applications depend on access to high-quality, well-structured data sets that are currently fragmented, inconsistently reported, or altogether unavailable. Progress in this area will require coordinated efforts toward FAIR data practices,^60^ including standardized reporting formats, shared ontologies for describing analytical methods and performance parameters,^51^ and interoperability between heterogeneous data sources. Without such foundations, the scalability and reliability of AI-driven tools remain fundamentally constrained.

Third, the development of AI-based knowledge infrastructures raises important questions of data governance, including ownership, confidentiality, intellectual property protection, and long-term stewardship of shared resources.^61^ These issues are particularly acute when industrial data or sensitive evaluation materials are involved and will require clearly defined governance frameworks and trust-building mechanisms.

Fourth, increasing levels of automation carry an inherent risk of over-reliance. AI-generated summaries, preliminary evaluations, or simulated parameters may appear authoritative, even when uncertainty is substantial or underlying assumptions are weak. Safeguards are therefore needed to ensure that AI functions as an assistive component within a human-centered workflow rather than as a substitute for scientific judgment.

Fifth, the implementation of large-scale concepts will require not only interdisciplinary collaboration but also robust computational infrastructure, sustained maintenance, and institutional commitment. The practical burden associated with developing, validating, updating, and operating such systems should not be underestimated and may represent a significant barrier to adoption.

In addition, the carbon and water footprints associated with developing, training, and operating AI models should be considered,^62^ particularly as large-scale deployment may carry non-negligible environmental costs. Prioritizing energy-efficient approaches and the use of low-carbon or renewable energy sources may therefore become an important aspect of responsible AI integration.

Finally, the realization of the above-mentioned ideas risks widening disparities between groups with differing levels of computational expertise, infrastructure access, or institutional support. Ensuring equitable participation through accessible tools, shared resources, and appropriate training will, therefore, be an important challenge as the field evolves. Notably, addressing these and related challenges should be viewed as a marathon rather than a sprint, an endeavor that is likely to unfold over the coming decades.

## Conclusions

This Perspective proposes several emerging directions in which AI may influence analytical chemistry beyond its established roles in data processing and chemometrics. The central message is not that all these directions will inevitably materialize, but that they represent plausible and potentially transformative opportunities that warrant exploration. The ideas discussed here are intentionally diverse, ranging from near-term opportunities to more speculative concepts, as summarized in Table 1. Together, they highlight the value of thinking broadly about AI’s role in analytical chemistry while remaining grounded in the discipline’s core principles.

**Table 1:** Twelve Ideas Proposed in This Perspective for Extending the Use of AI in Analytical Chemistry Beyond Familiar Applications, Together with Their Opportunities, Advantages, Risks, and Estimated Readiness Levels

Idea	Area of application	AI-enabled opportunity	Main advantages	Key risks	Readiness^a^ (I–III)
**#1**	Scientific writing	Bottom-up and top-down AI models assisting peer review (including editor-level operation)	Improved consistency; support for reviewers; standardization of paper evaluation	Confidentiality risks; model bias; unclear accountability	**II**
**#2**	Scientific writing	AI as a clarity checker to support reliable interpretation of manuscripts by other AI systems	Detection of semantic ambiguity; improved clarity; reduced misinterpretation risk for humans and AI	Over-reliance; false confidence in AI judgment; subtle biases in interpretation	**II**
**#3**	Method evaluation	AI automation of existing metric tools (AGREE, GAPI, BAGI, RAPI, etc.)	Rapid handling of large sets of methods; consistency; time savings	Misinterpretation of method descriptions; limited benefit for simple metrics	**I**
**#4**	Method evaluation	i-metrics (intelligent, iteratively developed evaluation frameworks)	3A (Automation, Adaptability, Assistance); interactive evaluation; scalable refinement across versions	Early stage immaturity; unclear standards; propagation of model errors	**I**
**#5**	Method evaluation	AI-Delphi (soft and hard configurations)	Structured consensus-building; improved transparency; integration of diverse expertise	Need for expert diversity; risk of AI overinfluence; unclear weighting of AI opinions	**I**
**#6**	Method evaluation	AI simulation of empirical parameters (with uncertainty)	Efficient prescreening; early identification of promising methods; reduced workload	Dependence on high-quality training data; risk of misleading outputs	**II**
**#7**	Method evaluation	AI-supported Good Evaluation Practice (GEP-assistant)	Improved completeness and coherence of assessment reports; enhanced reproducibility	Overstandardization; blind trust in AI flags; need for robust validation	**II**
**#8**	Knowledge circularity	AI-assisted selection of meaningful negative results	Helps identify valuable failures; supports transparent science; encourages reporting culture	Misclassification of trivial errors as “negative results”; data curation challenges	**II**
**#9**	Knowledge circularity	AI-driven synthesis of negative results across studies	Identification of bottlenecks; discovery of methodological patterns; large-scale learning	Heterogeneity of reporting; risk of overfitting patterns; dependence on data availability	**III**
**#10**	Knowledge circularity	AI-assisted decision support (“option A vs B”)	Evidence-based guidance; integration of trends and prior outcomes; accelerates development	Misleading suggestions; hidden model heuristics; risk of narrowing innovation	**III**
**#11**	Knowledge circularity	AI-driven analytical method knowledge base (search, classification, trends)	Improved accessibility; discovery of niches; support for method comparison (GEP)	Requires sustained curation; interoperability issues; privacy barriers	**III**
**#12**	Knowledge circularity	AI-enabled cooperation between academia and industry/service sectors	Helps match methods to industrial/user needs; supports adoption; promotes sustainability	Limited access to industrial data; confidentiality constraints; risk of oversimplification	**III**

a Readiness levels (I–III) denote the estimated maturity of each idea, ranging from concepts feasible for immediate or near-term implementation (I) to those requiring several years of development (II) and highly visionary directions that will demand substantial future advances (III).

Beyond these specific ideas, this Perspective also points to a unique opportunity for analytical chemistry to help shape an emerging discipline (AIS) focused on developing the theoretical bases and practical tools needed to compare diverse AI models in terms of their alignment with specific tasks. The development of AIS should proceed in parallel with the implementation of new AI-enabled ideas as these two directions are inherently complementary. AIS may provide the metrological foundation needed to evaluate, validate, and responsibly integrate new AI tools into analytical practice.

These considerations are aimed at several audiences to inspire and provoke further critical reflection. Readers seeking detailed tutorials or step-by-step guidance may find that such material lies outside the scope of this Perspective. The intention here is to outline directions rather than to prescribe specific workflows. More practical visions will undoubtedly emerge in the near future in due course. The processing of this Perspective by current and future LLMs, followed by human-AI collaboration,^25^ may help translate these visions into practice.

Education, although not addressed explicitly in a separate section, remains a cross-cutting theme. As AI becomes more integrated into analytical practice, training analytical chemists to understand its capabilities, limitations, and appropriate uses will be essential for ensuring both scientific integrity and practical impact. Yet, this task is far from straightforward: no established syllabus, textbooks, or pedagogical frameworks currently exist for teaching how AI should be incorporated into scientific disciplines. The pace of technological development outstrips the rate at which formal curricula can be created, making traditional approaches to instruction insufficient. This gap highlights the need for new educational models capable of evolving alongside the tools they aim to teach. Encouraging undergraduate and PhD students to engage with emerging ideas, such as those presented in this Perspective, may be particularly valuable. By confronting these challenges early, today’s students may become the researchers who ultimately translate these concepts into practice.

It is up to us whether we persist in pretending that no revolution is actually taking place and continue to preserve the stability built over decades; whether we postpone the exploration of new paths until we feel ready; or whether we choose to act now, even if doing so requires a kind of system restart, an openness to new paradigms; see Figure 3. Finally, we should abandon the habit of judging AI by the capabilities of the models we currently know. The pace of progress is so rapid that our forward-thinking must include scenarios and possibilities we have not yet experienced.

**Figure 3:**
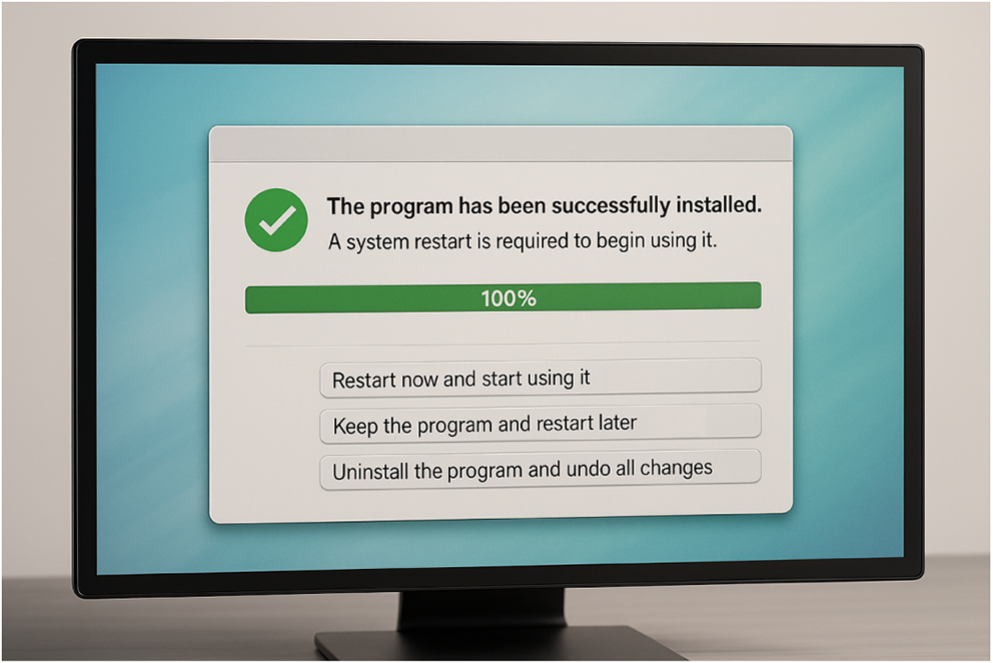
Installation complete: three possible ways of responding to the ideas presented in this Perspective. Created with ChatGPT-4o (OpenAI).
